# Kyasanur Forest Disease Virus: Epidemiological Insights, Pathogenesis, Therapeutic Strategies, and Advances in Vaccines and Diagnostics

**DOI:** 10.3390/v17081022

**Published:** 2025-07-22

**Authors:** Babita Bohra, Kumar Saurabh Srivastava, Ayush Raj, Nabanita Pal, Rahul Shukla

**Affiliations:** 1 Division of Virus Research and Therapeutics, CSIR-Central Drug Research Institute, Lucknow 226031, India; babitabohra0@gmail.com (B.B.); kssribt@gmail.com (K.S.S.); ayushraj.swork@gmail.com (A.R.); palnabanita13@gmail.com (N.P.); 2 Academy of Scientific and Innovative Research, Ghaziabad 201002, India

**Keywords:** Kyasanur Forest disease (KFD), KFD virus, tick-borne virus, zoonotic disease, public health, antiviral strategies

## Abstract

Kyasanur Forest disease virus (KFDV), a tick-borne Orthoflavivirus endemic to the Indian subcontinent, is a public health threat due to its recurrent outbreaks and expanding geographic range. This review provides a comprehensive overview of KFDV, encompassing its epidemiological trends, transmission dynamics, and ecological determinants that influence its spread. We delve into the current understanding of KFDV pathogenesis, highlighting key viral and host factors that drive infection and disease progression. Despite the absence of targeted antiviral therapies, recent advances have spurred the development of candidate therapeutics, including broad-spectrum antivirals and immunomodulators. We also discuss progress in vaccine development, with an emphasis on the limitations of the existing formalin-inactivated vaccine and the promise of next-generation platforms. Furthermore, we explore recent innovations in diagnostics, including molecular and serological tools, that aim to improve early detection and surveillance. A multidisciplinary approach integrating virology, immunology, ecology, and public health is essential for the effective management and eventual control of KFDV outbreaks.

## 1. Introduction

Kyasanur Forest disease virus (KFDV), a tick-borne Orthoflavivirus within the *Flaviviridae* family, causes a severe hemorrhagic fever known as Kyasanur Forest disease (KFD), primarily in India. The KFDV was first identified in 1957 in the Shimoga district of Karnataka. It is transmitted by *Haemaphysalis spinigera* ticks in a zoonotic cycle involving small mammals and monkeys, with humans serving as dead-end hosts [1,2,3]. The disease presents with biphasic symptoms, including fever, headache, and hemorrhage, with 10–20% of cases progressing to neurological complications and a case fatality rate of 3–5% [4,5,6]. Following its initial identification in 1957, approximately 10,000 cases of KFD were reported through 2017, with notable epidemic episodes documented in 1957–1958 (681 cases), 1983–1984 (2589 cases), 2002–2003 (1562 cases), and 2016–2017 (809 cases) [7]. Recent data (2019–2024) indicate 700–900 additional cases [8,9], predominantly in Karnataka (e.g., Shivamogga, Chikkamagaluru), but with emerging transmission in Kerala, Tamil Nadu, Goa, and Maharashtra, driven by deforestation, climate factors, and increased human–tick contact [10,11].

KFDV poses a growing public health challenge due to its geographic expansion and limited control measures [7,9,12]. The current formalin-inactivated vaccine demonstrates 62.4% efficacy with two doses and 82.9% with boosters; however, waning immunity and production challenges limit its effectiveness and contribute to ongoing outbreaks [13,14]. Due to suboptimal protection, the Kerala government discontinued vaccination in October 2022 (Department of Health and Family Welfare, Karnataka Government). Ongoing research and development on vaccine candidates represent a significant advancement beyond the currently available formalin-inactivated vaccine, which has demonstrated limited and variable efficacy, even in potency tests. Novel approaches, including vesicular stomatitis virus (VSV)-based live-attenuated platforms [15,16], subunit vaccines utilizing the recombinant envelope domain III [17], and in silico-designed multi-epitope constructs, have demonstrated promising immunogenicity and protective efficacy in preclinical models [18]. Additionally, rational design strategies supported by immunoinformatics have enabled the identification of conserved and immunodominant epitopes with potential cross-protective capabilities against related flaviviruses, such as Alkhurma hemorrhagic fever virus [18,19,20]. These vaccine candidates, though largely in the early stages of development, highlight a strategic shift toward more targeted and immunologically robust interventions. Continued experimental validation, coupled with collaborative frameworks led by institutions such as the Indian Council of Medical Research (ICMR)-National Institute of Virology (NIV), will be critical to advancing these candidates toward clinical evaluation.

No specific antivirals are currently approved for KFDV, and supportive care remains the standard treatment approach. The promising repurposed drug candidate, NITD008 (a nucleoside analogue inhibitor, broadly used to treat flavivirus infections), has emerged through computational modeling and has shown in vitro activity against KFDV [21]. Nonetheless, in vivo studies are essential to validate its protective efficacy and advance the compound toward clinical application.

Current KFDV diagnostics are led by RT-PCR and ELISA. RT-PCR is most effective during the early viremic phase but is constrained by timing and the need for specialized infrastructure. However, ELISA requires appropriately timed paired sera. Cross-reactivity is primarily an issue with ELISA due to antigenic similarity with other flaviviruses. Recent advances, such as dry-down RT-PCR and point-of-care (POC) devices, offer hope for faster, field-based diagnosis [22]. Further efforts are being made on multiplex assays, scalable technologies, and integrated surveillance to improve accuracy and accessibility, particularly for resource-poor settings [23].

Surveillance gaps, such as inconsistent case reporting and limited animal monitoring, further complicate outbreak response. These factors, combined with KFDV’s potential for cross-border spread and climate-driven tick dispersal [11,24], underscore the need for a comprehensive understanding of its epidemiology, pathogenesis, and control strategies (National Public Health India Conference, 2024. https://ncdc.mohfw.gov.in/wp-content/uploads/2024/03/E-Technodoc-_V9.pdf (accessed on 14 May 2025)

This review emphasizes current knowledge on KFDV, addressing five key areas: epidemiology (recent trends and regional spread), pathogenesis (molecular and immune mechanisms), therapeutics (repurposed drugs and gaps), vaccines (current and novel candidates), and diagnostics (advances and limitations). By integrating recent KFDV case data (2019–2024) and drawing on molecular, clinical, and public health studies, we aim to highlight research progress and gaps. This review also explores future directions, such as drug repurposing, next-generation vaccines, and enhanced surveillance, to mitigate KFDV infection. As KFDV emerges as an important public health concern, this review offers critical insights for researchers, policymakers, and public health practitioners.

## 2. KFDV Epidemiology

**Figure 1:**
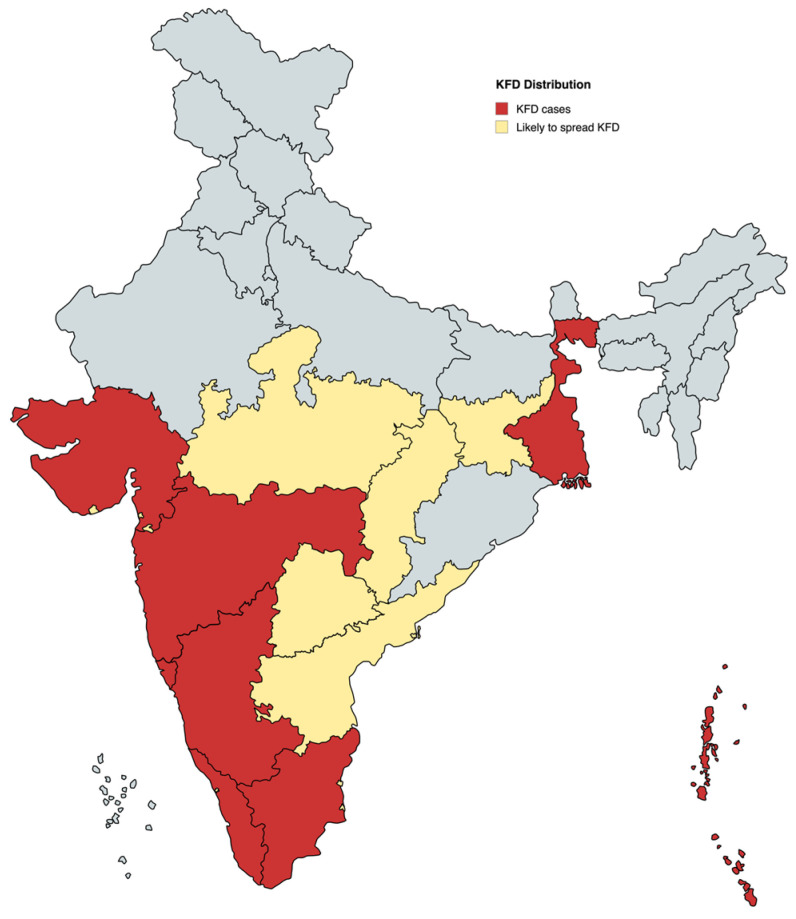
Geographic distribution and potential spread of KFDV in India. The map illustrates the current distribution of KFD cases (red) and states considered at risk for future spread (yellow), based on ecological suitability, tick vector prevalence, and forest coverage. The Western Ghats region, highlighted in red across states such as Karnataka, Kerala, Tamil Nadu, Goa, and Maharashtra, represents the primary endemic zone. States shown in yellow indicate regions with potential for viral emergence due to ecological continuity and increasing human–tick interactions. Data reflect KFDV distribution trends as of 2024.

KFDV is primarily endemic to the Western Ghats region of India, with cases reported in Karnataka, Tamil Nadu, Kerala, Goa, and Maharashtra. Since its first detection, the virus has exhibited significant geographical expansion beyond its endemic foci [10], with approximately 10,000 human cases documented, reflecting its persistence and spread into new ecological niches [7]. The virus’s spread is largely driven by factors such as deforestation, anthropogenic intrusion into forest habitats, and changes in vector and host dynamics [10]. The emergence of cases in neighboring states such as Kerala, Tamil Nadu, Goa, Maharashtra, and Gujarat underscores the evolving epidemiology of KFDV and highlights the need for robust surveillance, vector control, and public health interventions in the expanding endemic zones (Figure 1) [10,25,26,27]. Annual case numbers fluctuate, with 100–500 reported in peak years. Outbreaks occur mainly during dry months (December–May), coinciding with peak tick activity and human activity in forested areas [28]. Studies from Shivamogga, Karnataka, have noted inconsistent case reporting, complicating incidence estimates [29,30]. Seroprevalence studies suggest underreporting, as asymptomatic or mild cases may go undetected [12]. Recent data (2019–2024) suggest that the range of KFDV has expanded, with cases reported from different parts of Maharashtra and Gujarat states [8,31]. Cross-border spread to neighboring countries is a concern, although direct evidence is currently lacking. Enhanced surveillance and One Health Approaches are essential to curbing the public health impact of KFDV [12].

### 2.1. Transmission of KFDV

**Figure 2:**
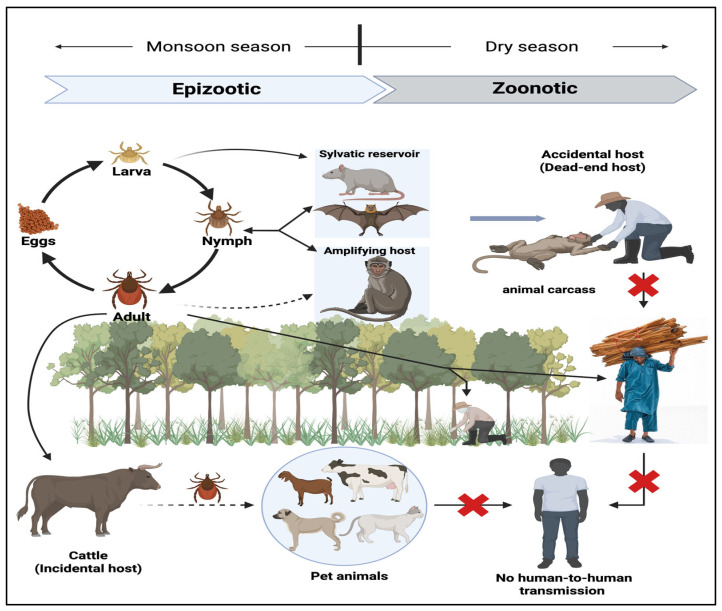
Ecology and transmission cycle of KFDV. The ixodid tick *Haemaphysalis spinigera* serves as both the principal reservoir and vector for KFDV. Infected ticks maintain lifelong viral carriage and can transmit the virus transovarially to progeny. The enzootic cycle involves small mammals and primates, which act as amplifying hosts; infection in primates often results in epizootics characterized by high mortality. Although larger domesticated animals (e.g., cattle, goats, and sheep) may acquire KFDV, they are considered incidental hosts with minimal contribution to human transmission dynamics. Pet animals may acquire the virus from the infected ticks and facilitate tick dispersal. During the dry season, zoonotic spillover occurs, with humans becoming accidental (dead-end) hosts, typically through exposure to infected ticks or contact with infected animal carcasses. Individuals engaged in forest-related occupational or recreational activities are at elevated risk of exposure. Red crosses indicate blocked transmission pathways.

KFDV is transmitted through the bites of infected ticks, primarily *Haemaphysalis spinigera* [3,32]. The virus circulates in a zoonotic cycle involving the small mammals (e.g., rodents, shrews), monkeys (e.g., *Macaca radiata* and *Semnopithecus priam*), and ticks. Monkeys, although considered amplifying hosts, experience high mortality and are thus regarded as sentinel species rather than true reservoir hosts. Large domestic animals such as goats, cows, and sheep exhibit low viremia and play a limited role in direct virus transmission. However, they act as blood sources for ticks and may contribute to the maintenance and spread of the virus among tick populations (Figure 2). Humans become infected accidentally and serve as dead-end hosts, with no confirmed evidence of human-to-human transmission to date. Occupational exposure (e.g., forest workers, farmers, hunters, and tribal populations) increases risk, particularly near sites or areas with dead monkeys. A case–control study conducted in Shivamogga (2022) identified the proximity to forested areas and dead monkey sites as major risk factors for disease transmission. Other contributing factors include the lack of protective clothing, low vaccination coverage, and environmental changes such as deforestation, which increase tick-human interactions. Children and adults in rural settings are equally affected, with no clear gender bias [31].

### 2.2. KFD Virus

**Figure 3:**
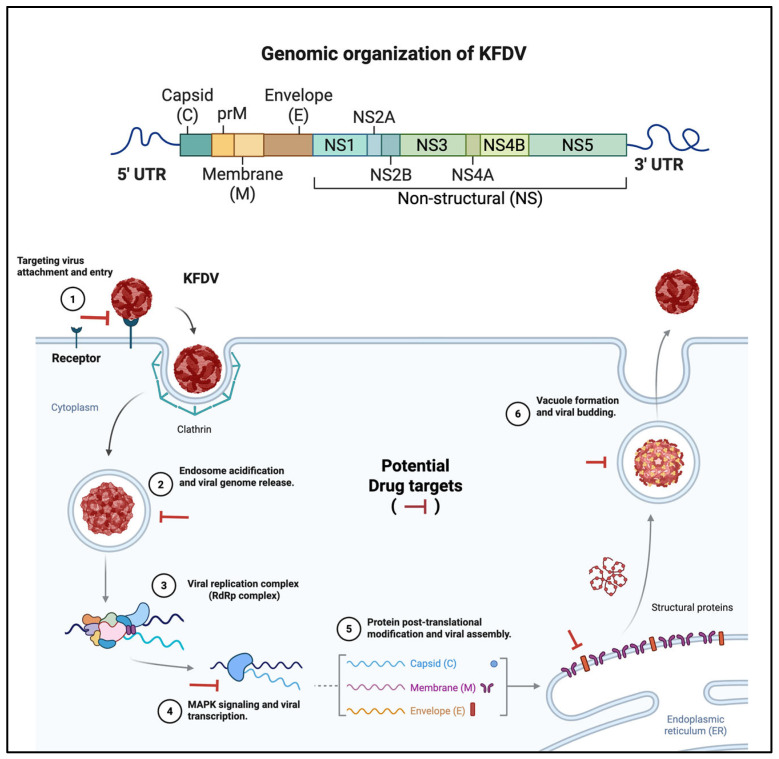
Genome organization and replication cycle of KFDV with potential antiviral targets. The top panel illustrates the genomic structure of KFDV, a positive-sense single-stranded RNA virus, comprising a single open reading frame flanked by 5’ and 3’ untranslated regions (UTRs). The genome encodes three structural proteins—capsid (C), premembrane (prM)/membrane (M), and envelope (E)—followed by seven nonstructural (NS) proteins (NS1, NS2A, NS2B, NS3, NS4A, NS4B, and NS5), involved in viral replication and immune evasion. The bottom panel depicts the intracellular life cycle of KFDV and highlights candidate stages for therapeutic intervention. (1) Viral attachment and entry occur via receptor-mediated endocytosis, facilitated by clathrin-coated vesicles. (2) Acidification of the endosome triggers viral uncoating and genome release into the cytoplasm. (3) The viral RNA is translated and processed to form the replication complex, including the RNA-dependent RNA polymerase (RdRp). (4) Host MAPK signalling pathways are hijacked to enhance viral transcription. (5) Structural proteins undergo post-translational modifications and are assembled in the endoplasmic reticulum (ER). (6) Mature virions bud off through vacuolar transport. Red inhibitory bars indicate putative antiviral drug targets at critical stages of the viral replication cycle, including entry, genome release, RNA replication, protein processing, and virion assembly.

KFDV is an enveloped, single-stranded, positive-sense RNA virus belonging to the *Flaviviridae* family. It has an ~11 kb genome and encodes a single polyprotein that is cleaved into three structural proteins—capsid (C), premembrane/membrane (prM/M), and envelope (E), and seven nonstructural (NS) proteins (NS1, NS2A, NS2B, NS3, NS4A, NS4B, NS5) (Figure 3). Like other flaviviruses such as dengue virus (DENV) and Zika virus (ZIKV), KFDV-E protein mediates cell entry and is a key target for neutralizing antibodies following viral infection, while C is the primary structural protein of the virus, which surrounds the viral genome. Premembrane (prM) is a glycosylated precursor protein to the small transmembrane protein M, which primarily facilitates viral maturation and entry into the host cell. Similar to DENV, NS1 plays a critical role in immune evasion and induces oxidative stress by activating antioxidant defences. NS1-specific antibodies (Abs) may contribute to vascular leakage by disrupting the endothelial glycocalyx, as observed in DENV infection, and may play a key role in immune responses that influence vascular permeability. However, the exact mechanisms and implications of NS1-specific antibodies are still under investigation. NS2A and NS2B, along with NS4A and NS4B, participate in viral replication and pathogenesis. NS3 contains both a serine protease domain (NS3pro) and a helicase domain (NS3hel), which are essential for viral replication and maturation [33]. NS5, possessing RNA-dependent RNA polymerase and methyltransferase activities, plays a central role in viral replication and also inhibits host–interferon (IFN) responses [34,35].

### 2.3. KFDV Pathogenesis

Following a bite from an infected *Haemaphysalis spinigera* tick, KFDV starts replicating in the host’s skin dendritic cells before disseminating to lymphoid tissues, liver, spleen, and, in severe cases, the brain. Viremia typically develops within 3–8 days and correlates with the initial febrile phase and systemic symptoms [36]. The KFDV tropism in hepatocytes and endothelial cells contributes to hemorrhagic manifestations, while neuroinvasion, as observed in 10–20% of cases, can lead to meningitis or encephalitis, corresponding to the second or “toxic” phase of illness [36,37]. A recent study in bonnet macaques detected viral RNA in the spleen, liver, and brain, with only 20% developing severe disease, suggesting that host factors modulate disease outcomes [38]. The molecular mechanisms underlying KFDV neuroinvasiveness remain poorly understood.

In terms of host–pathogen interactions, KFDV employs multiple strategies to evade host immune defences. The NS5 protein inhibits IFN-α/β signalling by suppressing STAT2 phosphorylation, similar to other flaviviruses such as DENV and ZIKV [39]. NS3, a protease–helicase, facilitates polyprotein cleavage and viral replication, while NS1 disrupts complement activation [40], thereby enhancing systemic spread. Studies have noted that mutations in KFDV’s envelope (E) protein may alter glycosaminoglycan binding, similar to other flaviviruses, and may increase infectivity in endothelial or neuronal cells [36,41,42]. Host factors, such as polymorphisms in innate immune genes (e.g., TLR3, IFITM3), may influence disease severity, although no KFDV-specific studies have yet explored this. In contrast to Alkhurma hemorrhagic fever virus (AHFV), as a KFDV variant that causes lethal disease in IFNAR^−/−^ mice, KFDV pathogenesis in immunocompromised models like AG129 mice remains untested, limiting insights into IFN-dependent mechanisms.

KFDV infection elicits robust innate and adaptive immune responses, though these are often delayed or insufficient to prevent severe disease. Devadiga et al. (2020) [43] reported that in human KFD patients, early activation of CD8+ T-cells and natural killer (NK) cells was observed, peaking 7–10 days post-infection, along with elevated levels of proinflammatory cytokines (specially IL-6, TNF-α), which correlate with fever and hemorrhagic manifestations. B-cell responses produce IgM within 5–7 days, followed by IgG, which typically coincides with virus clearance. Prior vaccination enhances IgG titers, reducing disease severity. In bonnet macaques, 80% of infected animals cleared the virus without severe symptoms, suggesting effective Th1-mediated immunity in some hosts. However, immune evasion by KFDV, particularly via NS5-mediated IFN suppression, may allow prolonged viremia in severe cases. The role of antibody-dependent enhancement (ADE), as observed in DENV, remains unexplored in KFDV but warrants investigation given the similarities among flaviviruses.

KFDV shares structural and pathogenic features with DENV, ZIKV, and tick-borne encephalitis virus (TBEV). Like DENV, KFDV causes hemorrhagic fever and targets endothelial cells; however, its neuroinvasiveness more closely resembles that of TBEV. AHFV, a KFDV variant, exhibits higher lethality in IFNAR^−/−^ mice, possibly due to distinct NS3 or NS5 mutations, whereas KFDV’s neurovirulence may be attributable to passaging artefacts. Unlike DENV, which has well-established mouse models (e.g., AG129), KFDV pathogenesis studies primarily rely on BALB/c mice or non-human primates, limiting detailed molecular insights. The E protein’s role in receptor binding and immune evasion is conserved across flaviviruses; however, the specific host receptors for KFDV (e.g., DC-SIGN, TIM-1) remain unidentified.

Several gaps hinder a comprehensive understanding of KFDV molecular pathogenesis. First, the functional impact of E protein mutations on tropism and virulence requires validation through both in vitro and in vivo studies. Second, the lack of immunocompromised mouse models (e.g., AG129) for KFDV limits investigations into IFN-dependent pathogenesis and antiviral testing. Third, long-term sequelae, such as neurological deficits, remain poorly characterized, with no available data on viral persistent or immune memory. Fourth, the role of host genetic factors in disease susceptibility is largely unexplored. Future research should prioritize high-resolution structural studies of KFDV proteins, single-cell RNA sequencing of infected tissues, and the development of KFDV-specific animal models to elucidate molecular mechanisms and guide therapeutic design.

### 2.4. Therapeutic Options for KFDV

KFDV currently lacks specific antiviral therapies, making supportive care the cornerstone of clinical management. In this section, we review the current landscape of KFDV therapeutics, assess the status of repurposed drugs with potential efficacy, identify key challenges, and propose future directions to address this critical gap in KFDV control.

### 2.5. Current Status

To date, no FDA-approved or clinically validated antiviral agents are available for KFDV. Management remains supportive, relying on symptomatic treatment such as hydration, antipyretics, and (in severe cases) blood transfusions or intensive care for hemorrhagic and neurological complications. The case fatality rate of 3–5% and the biphasic progression of the disease highlight the need for stage-specific therapeutics and preventive interventions. The absence of specific therapeutics exacerbates public health challenges, especially in endemic regions like the Shivamogga and Chikkamagaluru districts of Karnataka, where outbreaks continue despite vaccination efforts.

### 2.6. Repurposed Drugs

Given the lack of KFDV-specific drug development, the repurposing of existing antivirals offers a promising strategy. Several candidates have been explored or hypothesized based on activity against related flaviviruses (Table 1):

NITD008: A nucleoside analog inhibitor, NITD008, has demonstrated in vitro activity against KFDV and other flaviviruses such as DENV and ZIKV by blocking RNA synthesis [21]. However, preclinical toxicity in animal models has halted further development [44], highlighting the need for safer analogs.

Favipiravir and Sofosbuvir: These broad-spectrum antivirals effective against RNA viruses, including Ebola virus [45] and hepatitis C virus [46], have shown in vitro efficacy against multiple flaviviruses [47,48,49,50,51]. Although not tested specifically for KFDV, their mechanisms of action, such as RNA-dependent RNA polymerase inhibition [51], suggest potential and warrant investigation in KFDV models.

Niclosamide: An FDA-approved anthelmintic, Niclosamide, has shown antiviral activity against DENV both in vitro and in vivo, reducing viral replication and viremia [52]. Whilst no studies have evaluated Niclosamide against KFDV, its ability to inhibit flavivirus replication via autophagy induction [53] and NS2B-NS3 protease disruption [54] makes it a viable repurposing candidate. Clinical trials would be required to assess its efficacy and safety against KFDV.

**Table 1:** Drug candidates for therapeutics.

Therapeutic Agent	Mechanism of Action	Evidence of Activity	Current Status	Notes for KFDV Potential
NITD008	Nucleoside analog; inhibits RNA synthesis	In vitro activity against KFDV, DENV, Zika [21,44,55]	Preclinical; discontinued due to toxicity	Demonstrated KFDV inhibition; safer analogs needed; AG129 model testing pending.
Favipiravir	RNA-dependent RNA polymerase inhibitor	In vitro/vivo activity against Ebola, DENV [56,57]	Clinically approved (e.g., influenza); not tested for KFDV	Broad-spectrum; potential for KFDV NS5 targeting; clinical trials required.
Sofosbuvir	NS5B polymerase inhibitor	In vitro activity against HCV, DENV [46,58]	Clinically approved (HCV); not tested for KFDV	Flavivirus cross-reactivity possible; in vivo KFDV studies needed.
Niclosamide	Induces autophagy; inhibits NS2B-NS3 protease	In vitro and in vivo activity [52,53] against DENV and SARS-CoV-2 [59]	FDA-approved (anthelmintic); not tested for KFDV	Promising for KFDV due to flavivirus similarity; AG129 model adaptation suggested.
Monoclonal Antibodies	Neutralizes E protein; blocks viral entry	Effective in DENV passive immunization [60]	Preclinical/early clinical for DENV; none for KFDV	KFDV-specific antibodies needed; risk of cross-reactivity with other flaviviruses.

### 2.7. Challenges in Developing Therapeutics for KFDV

Several obstacles hinder therapeutic advancement for KFDV. First, the lack of validated animal models limits preclinical testing. While AG129 mice are widely used to test antivirals against DENV and ZIKV, to date, no studies have adapted this model for KFDV, leaving uncertainty about IFN-dependent pathogenesis and drug efficacy. Second, high-throughput screening for KFDV-specific antivirals is constrained by limited funding and a lower research priority compared to other flaviviruses. Third, the biphasic nature of KFD, with neurological complications in 10–20% of cases, complicates therapeutic timing and delivery. Finally, the geographic isolation of endemic areas (e.g., the Western Ghats) and lack of healthcare infrastructure pose significant logistical challenges to conducting clinical trials and implementing effective vaccination strategies.

To address these challenges, a multifaceted approach is required. First, developing KFDV-specific animal models, such as AG129 or IFNAR^−/−^ mice, would enable rigorous testing of repurposed drugs like Niclosamide, Flavipiravir, and Sofosbuvir. Second, high-throughput screening of approved antiviral libraries, targeting KFDV NS3, NS5 and E proteins, could uncover novel candidates. Third, combination therapies, pairing nucleoside analogs with monoclonal antibodies, may enhance efficacy and reduce resistance, as demonstrated in DENV studies [61,62]. Fourth, leveraging computational drug discovery methods (e.g., molecular docking of KFDV NS3 protease, helicase, and NS5 RdRp inhibitors) could accelerate lead identification. Finally, clinical trials in endemic regions, supported by portable diagnostic tools, are essential for validating efficacy and ensuring equitable access. Collaborative efforts under a One Health framework—integrating human, animal, and environmental data—could streamline therapeutic development and deployment.

In brief, the absence of specific therapeutics for KFDV remains a critical barrier to effective disease control, with supportive care proving insufficient for severe cases. Repurposed drugs like NITD008, along with promising candidates like Niclosamide and Favipiravir, offer hope but require rigorous validation against KFDV. Overcoming current limitations through the development of advanced animal models, screening platforms, and clinical trial infrastructure will be pivotal. As the geographic range of KFDV continues to expand, investing in targeted therapeutic research is imperative to reduce morbidity and mortality and to complement ongoing vaccine and diagnostic initiatives.

### 2.8. Vaccines

Despite the availability of a formalin-inactivated vaccine, its limited efficacy has prompted ongoing research into novel vaccine candidates. This section reviews the currently available vaccine, emerging immunization technologies, existing challenges, and future directions aimed at enhancing KFDV vaccination strategies.

### 2.9. Available Vaccine for KFDV

The formalin-inactivated KFDV vaccine, developed in India and in use since the 1960 s, remains the primary preventive measure against KFD. It is produced from mouse brain-passaged virus and requires two doses (a primary and a booster) followed by an annual booster. Studies [14,62] have reported an efficacy of 62.4% with two doses and 82.9% with booster doses, based on historical outbreak data. However, vaccine-induced protection wanes over time, with seroconversion rates dropping to 30–40% within a year [14,63], contributing to persistent outbreaks (>150 cases in 2023). In addition to limited efficacy, production challenges, including inconsistent antigen yield and reliance on mouse brains, have led to supply shortages, culminating in suspension of vaccine administration in Karnataka in October 2022. Vaccine hesitancy, driven by concerns over suboptimal efficacy and mild side effects (e.g., fever, local swelling), further limits coverage in endemic regions.

### 2.10. Novel Vaccine Candidates

Recent advancements have introduced promising alternatives to address the limitations of the current KFDV vaccine, detailed as follows (Table 2).

Vesicular Stomatitis Virus (VSV)-Based Vaccine: Bhatia et al. (2023) [15] developed a recombinant VSV vector expressing the KFDV E-protein, which demonstrated complete protection in BALB/c mice against lethal challenge. The vaccine elicited robust humoral immunity, with neutralizing antibodies detectable within 14 days. In follow-up studies, the same group [16] extended these findings to non-human primates (pig-tailed macaques), observing reduced viral loads, absence of severe disease, and cross-protection against AHFV, a KFDV variant. This VSV-platform allows for rapid production of a vaccine candidate and has well-established safety profile (as used in Ebola vaccines [64]), making it a leading candidate for clinical translation.

Multi-Epitope Subunit Vaccine: Arumugam et al., 2021 [18] employed an in silico approach to designing a subunit vaccine targeting conserved epitopes on the KFDV E-protein. The candidate was predicted to bind Toll-like receptor 2 (TLR-2) and elicited strong B-cell and T-cell responses in silico, with potential cross-protection against AHFV. Although not yet tested in vivo, its computational optimization suggests a cost-effective, scalable option for developing a multi-epitope vaccine against KFDV.

Other Platforms: mRNA and live-attenuated vaccines, which have shown success against other flaviviruses (e.g., ZIKV), have not yet been explored for KFDV. These platforms offer the advantage of rapid development and adaptability to emerging strains, but substantial investment and infrastructure are required to develop and deploy a KFDV-specific vaccine using these technologies.

**Table 2:** Vaccine candidates for KFDV.

Vaccine Name	Platform/ Technology	Evidence of Efficacy	Current Status	Notes for KFDV Potential
Formalin-Inactivated Vaccine	Inactivated whole virus (mouse brain-derived)	62.4% efficacy (2 doses), 82.9% (with boosters) [14,43]	In use since the 1960s; suspended in 2022	Partial protection; waning immunity; production challenges; booster dependency.
VSV-Based Vaccine	Recombinant vesicular stomatitis virus (VSV) expressing KFDV E protein	100% protection in BALB/c mice [16]; reduced viral load in macaques [15]; cross-protects against AHFV	Preclinical (mice, macaques)	Promising efficacy and safety; Phase I/II trials needed; scalable production is potential.
Multi-Epitope Subunit Vaccine	Recombinant subunit (in silico designed E protein epitopes)	Strong B/T-cell responses predicted in silico; binds TLR-2 [18]	Preclinical (in silico)	Cost-effective; in vivo validation pending; potential AHFV cross-protection.
mRNA Vaccine	mRNA encoding KFDV antigens	Effective for Zika, SARS-CoV-2 [65,66,67]	None of the study done for KFDV	Rapid development potential; adaptable to strains; requires KFDV-specific design.
Live-Attenuated Vaccine	Attenuated KFDV strain	Successful for yellow fever [68] 2017, DENV [69]	Not developed for KFDV	Could induce robust immunity; safety concerns need addressing; preclinical testing needed.

### 2.11. Challenges in KFDV Vaccination and Development

Several obstacles impede progress in KFDV vaccine development. First, the suboptimal efficacy and production limitations of the current vaccine highlight the need for standardized immunogenicity assays and scalable manufacturing platforms. Second, limited clinical trial data—as most studies are preclinical—delay regulatory approval and widespread deployment. Third, the biphasic nature of KFDV, with neurological complications, complicates vaccine safety assessments, particularly in immunocompromised individuals. Fourth, geographic and logistical barriers in endemic regions of Western Ghats hinder vaccine distribution, delivery, and post-vaccination monitoring. Finally, the lack of a defined correlate of protection (e.g., specific antibody titers) complicates the evaluation of vaccine efficacy, as evidenced by the variable performance of the formalin-inactivated vaccine.

To overcome these challenges, a multifaceted strategy is essential. First, advancing the VSV-based vaccine to Phase I/II clinical trials in endemic populations (e.g., Karnataka, Kerala) should be prioritized, leveraging its demonstrated cross-protective potential against AHFV. Second, validating the multi-epitope subunit vaccine in relevant animal models (e.g., bonnet macaques) and initiating early-phase human trials could diversify the vaccine pipeline. Third, exploring mRNA and live-attenuated platforms tailored to KFDV’s genetic diversity may enhance adaptability and long-term efficacy. Fourth, integrating vaccine development with One Health approaches, such as immunizing non-human primates (e.g., red-faced bonnet monkeys) to reduce zoonotic reservoirs, could strengthen community-level immunity (herd immunity). Fifth, establishing long-term immunogenicity studies and identifying correlates of protection will be critical to guiding booster schedules and regulatory benchmarks. Finally, public health campaigns that address vaccine hesitancy, supported by real-world evidence from clinical trials, will be essential to ensure widespread vaccine uptake in affected regions.

In a nutshell, the formalin-inactivated KFDV vaccine provides partial protection but remains insufficient to control outbreaks, highlighting the need for innovative alternatives. The VSV-based and multi-epitope subunit vaccines represent significant advancement with promising clinical potential. Overcoming challenges related to production, safety, and distribution (through advanced modern technologies and integrated strategies) will be essential. As KFDV’s geographic range expands, accelerating vaccine development is critical to reducing the disease burden and should complement parallel efforts in therapeutics and diagnostics.

## 3. Diagnostics

Accurate and timely diagnosis of KFDV is essential for managing its rapidly widespread incidence in India, particularly in endemic regions of Karnataka. Given the notably high case fatality rate and the potential for neurological complications in 10–20% of cases, robust diagnostic tools are critical for guiding clinical management and controlling outbreaks. This section reviews current diagnostic methods, recent technological advancements, persistent challenges, and future directions aimed at enhancing KFDV detection.

### 3.1. Current Diagnostic Tools

The gold standard for KFDV diagnosis is reverse transcription polymerase chain reaction (RT-PCR), which detects viral RNA in serum or tissue during the acute phase (typically days 3–8 post-infection). Yadav et al., 2023 [22] standardized a dry-down RT-PCR assay targeting the KFDV envelope gene, demonstrating high sensitivity (95%) and specificity (98%) for early detection. While this method is well suited for field deployment, it still requires trained personnel and basic laboratory infrastructure.

Serological assays, such as enzyme-linked immunosorbent assay (ELISA), complement RT-PCR by detecting IgM and IgG antibodies. Rajak et al., 2022 [70] described the development of a recombinant envelope domain III (EDIII)-based ELISA, achieving 92% sensitivity and 94% specificity for IgM detection within 5–7 days post-onset, thereby supporting diagnosis during the second phase of the disease. Rapid diagnostic tests (RDTs) based on antigen detection are currently under development, but none have been validated for use in KFDV to date.

The Plaque Reduction Neutralization Test (PRNT) is widely considered the gold standard for serological confirmation for flavivirus infections, including KFDV. Although the PRNT is not routinely used in clinical diagnostic settings due to its labour-intensive protocol, requirement for live virus, and need for biosafety level-3 (BSL-3) containment facilities, it remains indispensable for confirmatory diagnosis.

A summary of available diagnostic tools is provided in the Table 3.

### 3.2. Challenges in KFDV Diagnosis

Several challenges hinder the effective diagnosis of KFDV. First, the narrow diagnostic window; RT-PCR is effective only during the viremic phase (days 3–8 post-infection), while ELISA relies on seroconversion (typically days 5–14) and can result in missed cases outside these periods, contributing to underreporting, including asymptomatic infections observed in regions such as Kerala. Second, limited infrastructure in rural areas, such as a lack of cold chains for reagent storage and a shortage of trained staff, restricts the implementation of RT-PCR and ELISA. Third, serological cross-reactivity with other flaviviruses remains a significant concern, particularly for ELISA-based assays, complicating differential diagnosis in co-endemic regions. Fourth, the high cost and technical complexity of NGS currently limit its feasibility for routine surveillance. Finally, the biphasic clinical nature of KFDV, with neurological symptoms typically emerging later, delays suspicion and testing.

To address these challenges, several advancements are necessary. First, the development of multiplex RT-PCR or POC assays capable of differentiating KFDV from DENV, ZIKA, and Japanese encephalitis viruses would significantly enhance diagnostic specificity and turnaround time. Second, scaling up dry-down RT-PCR platforms and validating POC devices for use in endemic regions and other potentially affected states could help reduce diagnostic delays. Third, integrating artificial intelligence (AI) with NGS data could improve strain tracking and enable the prediction of outbreak hotspots. Fourth, affordable, thermostable RDTs targeting KFDV antigens or antibodies, potentially based on lateral flow technology, are critical for deployment in rural settings. Fifth, the establishment of standardized diagnostic algorithms that combine RT-PCR, ELISA and serological testing would optimize case detection across the different phases of the disease. Finally, implementing training programs for local health workers and deploying mobile diagnostic units would help to bridge infrastructure gaps and support a comprehensive One Health surveillance framework.

## 4. Discussion

KFDV has emerged as a persistent health threat, with approximately 700–900 cases reported across India between 2019 and 2024 [31], predominantly from the Shivamogga and Chikkamagaluru districts of Karnataka. The virus has also expanded into Kerala, Tamil Nadu, Goa, and Maharashtra, raising concern about potential spread into neighboring states like Andhra Pradesh, Telangana, and Madhya Pradesh. This review consolidates critical insights into KFDV epidemiology, pathogenesis, therapeutics, vaccines, and diagnostics, revealing both recent progress and critical gaps that require urgent attention.

Epidemiologically, KFDV’s geographic spread, driven by deforestation and climate factors (e.g., heat, low rainfall, and humidity), highlights the need for robust surveillance. The 3–5% case fatality rate and neurological complications in 10–20% of cases underscore the disease’s severity, while underreporting (e.g., asymptomatic cases in Kerala) complicates incidence estimates. Molecular pathogenesis has revealed key mechanisms, such as NS5-mediated interferon suppression and adaptive E protein mutations, but validated immunocompromised models (e.g., AG129 mice) used to fully explore host–pathogen dynamics remain lacking. Therapeutically, the absence of specific antivirals leaves supportive care as the mainstay, with repurposed candidates like Niclosamide (effective against dengue) still untested for KFDV. Vaccines show promise, with the VSV-based and multi-epitope subunit approaches offering protection in preclinical models, but the 62.4% efficacy of the formalin-inactivated vaccine limits outbreak control. Diagnostics have advanced with dry-down RT-PCR, reducing turnaround time to 4–6 h, yet infrastructure gaps and cross-reactivity challenges persist.

### 4.1. Research Gaps

Several gaps continue to hinder effective KFDV management. In epidemiology, inconsistent surveillance (e.g., 2–16 days testing delays) and lack of standardized seroprevalence data obscure the true burden. Pathogenesis studies are constrained by the absence of KFDV-specific animal models, restricting insights into neuroinvasiveness and long-term sequelae. Therapeutics’ development is hampered by the lack of high-throughput screening and clinical trials, with no available data on drug efficacy in endemic populations. Vaccine research requires clinical validation and the identification of correlates of protection, while diagnostics demand multiplex assays to differentiate KFDV from co-endemic flaviviruses. These gaps collectively impede a cohesive and effective response to KFDV’s rising incidence.

### 4.2. Future Directions

Addressing these challenges requires a multifaceted approach. In epidemiology, implementing real-time surveillance systems with mobile diagnostic units and standardized reporting can enhance case detection, particularly in rural regions of the Western Ghats. Pathogenesis research should prioritize the development of AG129 or IFNAR^−/−^ mouse models to elucidate IFN-dependent mechanisms and evaluate therapeutic candidates. Therapeutics’ development can advance by repurposing drugs (e.g., Niclosavir, Favipiravir) through high-throughput screening targeting viral vital proteins such as NS2B-NS3 protease, NS3 helicase, NS5 and E proteins, followed by Phase I/II trials in endemic regions. Vaccine development should prioritize advancing the VSV-based candidate to clinical trials, exploring mRNA platforms for strain adaptability and integrating non-human primate immunization to reduce zoonotic reservoirs. Diagnostics require investment in affordable, thermostable point-of-care devices and AI-enhanced NGS for outbreak prediction, enabling phylogeographic studies. A One Health framework and integrating human, animal (e.g., red-faced bonnet monkeys), and environmental data can unify these efforts, supported by community education initiatives to address vaccine hesitancy.

### 4.3. Global Implications and Integrated Strategies

KFDV has potential risk for cross-border spread, as seen with Alkhurma hemorrhagic fever virus; this makes it a model for tick-borne flavivirus control. Climate-driven tick dispersal and deforestation amplify this risk, necessitating international collaboration. Integrated strategies, combining enhanced surveillance, robust drug and vaccine development pipelines, and diagnostic innovation, can transform KFDV management. Policymakers must prioritize sustained funding and infrastructure development, while researchers should focus on translational research to bridge preclinical and clinical gaps. As the KFDV burden increases, this holistic approach will not only mitigate its impact in India but also inform global responses to emerging flaviviral threats.

## 5. Conclusions

Kyasanur Forest disease virus remains a significant public health challenge, with recurrent outbreaks predominantly in Karnataka’s Shivamogga and Chikkamagaluru regions and emerging in Kerala, Tamil Nadu, Goa, and Maharashtra. Its 3–5% case fatality rate and neurological complications in 10–20% of cases underscore the disease’s severity, which is exacerbated by its expanding geographic range and climate-driven tick proliferation. This review highlights key insights; epidemiology reveals persistent outbreaks despite vaccination; pathogenesis studies have uncovered the molecular mechanisms but are hindered by the lack of robust models; therapeutics remain limited to supportive care, although repurposed candidates show promise; vaccines, notably VSV-based and subunit approaches have progressed but require clinical validation; and diagnostics have improved with dry-down RT-PCR, though challenges remain regarding turnaround time and infrastructure.

The existing gaps due to underreporting, lack of KFDV-specific antivirals, suboptimal vaccine efficacy, and diagnostic delays demand urgent action. Integrated strategies under a One Health framework—combining enhanced surveillance, drug repurposing, next-generation vaccines (e.g., mRNA), and field-deployable diagnostics—are essential. Collaborative efforts across research, policy, and community levels are crucial to accelerate progress, particularly in endemic regions of the Western Ghats. As the potential for KFDV’s global spread increases, investing in these areas will not only mitigate its burden but also inform control strategies for other tick-borne flaviviruses. This review calls for immediate, multidisciplinary action to shift KFDV from an emerging threat to a manageable disease.

## Figures and Tables

**Table 3:** Diagnostic Methods for KFDV.

Diagnostic Methods	Technique	Detection Target	Sensitivity/Specificity	Current Status	Notes on Limitations and Potential Improvements
RT-PCR (Standard)	Reverse transcription PCR	Viral RNA (envelope gene)	~95%/~98% [71]	Routine in labs	Limited to the viremic phase (days 3–8); requires infrastructure; dry-down version improves field use.
Dry-Down RT-PCR	Lyophilized RT-PCR	Viral RNA (envelope gene)	~95%/~98% [22]	Emerging (field testing)	Reduces turnaround to 4–6 h; needs validation in remote settings; scalable production needed.
ELISA (IgM/IgG)	Enzyme-linked immunosorbent assay	IgM/IgG antibodies	~92%/~94% (IgM) [70]	Routine in labs	Cross-reactivity with flaviviruses; delayed detection (days 5–14); enhances with recombinant antigens.
Next-Generation Sequencing (NGS)	High-throughput sequencing	Whole viral genome	Variable (research-grade [72]	Research tool	Costly and complex; not routine; potential for AI integration to track strains.
Point-of-Care (POC) Devices	Lateral flow or RT-PCR-based	KFDV Antigens or RNA	Under validation (~90% est.) [73]	Prototype (development)	Limited validation; needs thermostable, affordable design for rural deployment.

## Data Availability

No new data were created or analyzed in this study.
